# Acupuncture for chronic stable angina pectoris based on the theory of Meridian-Viscera Association: study protocol for a multicenter randomized controlled trial

**DOI:** 10.1186/s13063-020-04836-8

**Published:** 2020-11-06

**Authors:** Zhaoxuan He, Yi Yang, Qiao Wen, Tao Yin, Zhengjie Li, Peihong Ma, Hui Zheng, Yunhong Yang, Yongliang Jiang, Jianqiao Fang, Lei Lan, Fang Zeng

**Affiliations:** 1 Acupuncture and Tuina School/The 3rd Teaching Hospital, Chengdu University of Traditional Chinese Medicine, 37# Shierqiao Road, Chengdu, 610075 Sichuan China; 2 Acupuncture-Brain Research Center, Chengdu University of Traditional Chinese Medicine, Chengdu, Sichuan China; 3 School of Administration, Chengdu University of Traditional Chinese Medicine, Chengdu, Sichuan China; 4 Department of Neurobiology and Acupuncture Research/The Third Clinical Medical College, Zhejiang Chinese Medical University, Hangzhou, Zhejiang China; 5 Key Laboratory of Sichuan Province for Acupuncture & Chronobiology, Chengdu, Sichuan China

**Keywords:** Chronic stable angina pectoris, Acupoint, Randomized controlled trial, Intradermal needle, Meridian-Viscera Association

## Abstract

**Background:**

Acupuncture has long been used to treat chronic stable angina pectoris (CSAP), but the acupuncture prescription for CSAP varies. This trial will compare the therapeutic effects between the acupoints on the Heart Meridian and the acupoints on the Lung Meridian for treating CSAP, so as to provide a better prescription for acupuncture treatment of CSAP.

**Methods:**

This is a multicenter randomized controlled trial. A total of 148 CSAP patients will be randomly allocated into two groups through central randomization in a 1:1 ratio. This trial will include a 2-week screening period, a 4-week treatment period, and a 4-week follow-up period. The primary outcome is the frequency of angina attacks from baseline to 4 weeks after inclusion. Secondary outcomes include the frequency of angina attacks from baseline to 4 weeks after acupuncture treatment, the pain intensity of angina, total ischemia burden, heart rate variability, QT dispersion, the score of Seattle Angina Questionnaire, and the score of Short-Form of McGill Pain Questionnaire. These outcome measures will be evaluated at baseline, at the end of acupuncture treatment, and at the end of follow-up.

**Discussion:**

We hypothesize that the effectiveness of puncturing at acupoints on the Heart Meridian will not be the same as those on the Lung Meridian. The results will provide further evidence of Meridian-Viscera Association theory and references for acupoints selection in the clinical practice.

**Trial registration:**

Chinese Clinical Trial Registry ChiCTR1900025804. Registered on September 9, 2019

## Background

Chronic stable angina pectoris (CSAP) is a common manifestation of coronary artery disease (CAD) and serves as the initial symptom in approximately half of all patients with CAD [1–4]. In America, an estimated 15.5 million adults have chronic CAD, and more than 7 million adults suffer from angina [5]. CSAP is associated with an increased risk of major cardiovascular events and sudden cardiac death [6], significantly impacts the patients’ quality of life (Qol) [7], and increases the considerable healthcare expenditure [8]. Currently, the main management of CSAP is pharmacological treatment including β-blockers, angiotensin-converting enzyme inhibitors (ACEI), angiotensin II receptor blockers (ARB), and antiplatelet medications and statins [9]. However, the efficacy of medications is not satisfactory for the ignored side effects [2].

Acupuncture has been used for cardiovascular disorders including precordial pain and palpitation for thousands of years [10, 11]. The therapeutic effects of acupuncture for treating CSAP are gradually being accepted in Western counties. Recently, some randomized controlled trials (RCT) had investigated the clinic value of acupuncture for treating CSAP. For example, acupuncture as an adjunctive treatment to antianginal therapy could significantly alleviate angina compared to the waiting list [12]; acupuncture increased cardiac work capacity significantly compared to sham acupuncture, which expressed as dPRP (difference in pressure-rate-product between rest and maximum exercise) and maximal PRP during exercise [13]; and acupuncture was superior to isosorbide dinitrate and nifedipine in improving the symptoms of coronary angina pectoris [14]. Since these studies proved the efficacy of acupuncture for CSAP from different aspects, how to improve the effectiveness and accessibility of acupuncture for CSAP attracts increasing attention.

According to the traditional Chinese acupuncture theory, meridians and viscera are closely related in physiological function, mutually affected in pathological changes and treatment. The Heart Meridian connects with the heart directly, and the Lung Meridian indirectly associates with the heart. Both of these two meridians can be used for cardiovascular diseases in clinical practice [15, 16]. Based on the theory of Meridian-Viscera Association, our previous study found that not only the acupoints on the Heart Meridian and Pericardium Meridian, but also the acupoints on the Lung Meridian could improve the symptoms and the Qol of CSAP patients. To further optimize the acupoint prescription and provide more convenience and simple acupuncture therapy for CSAP, we designed this RCT. In this study, acupuncture prescription is composed of the basic acupoints (local acupoints close to the heart) and additional acupoints (acupoints on the Heart Meridian or the Lung Meridian), which are far from the heart. The aim of this study focuses on providing the reference for acupoint and acupuncture modality selection in acupuncture treating CSAP, so as to promote the application of acupuncture for treating CSAP. The intradermal needle has been selected as the acupuncture intervention for its advantages including continuous stimulation, high convenience, high safety, and non-invasiveness compared with the filiform needle [17, 18].

## Methods and design

### Study design

**Fig. 1:**
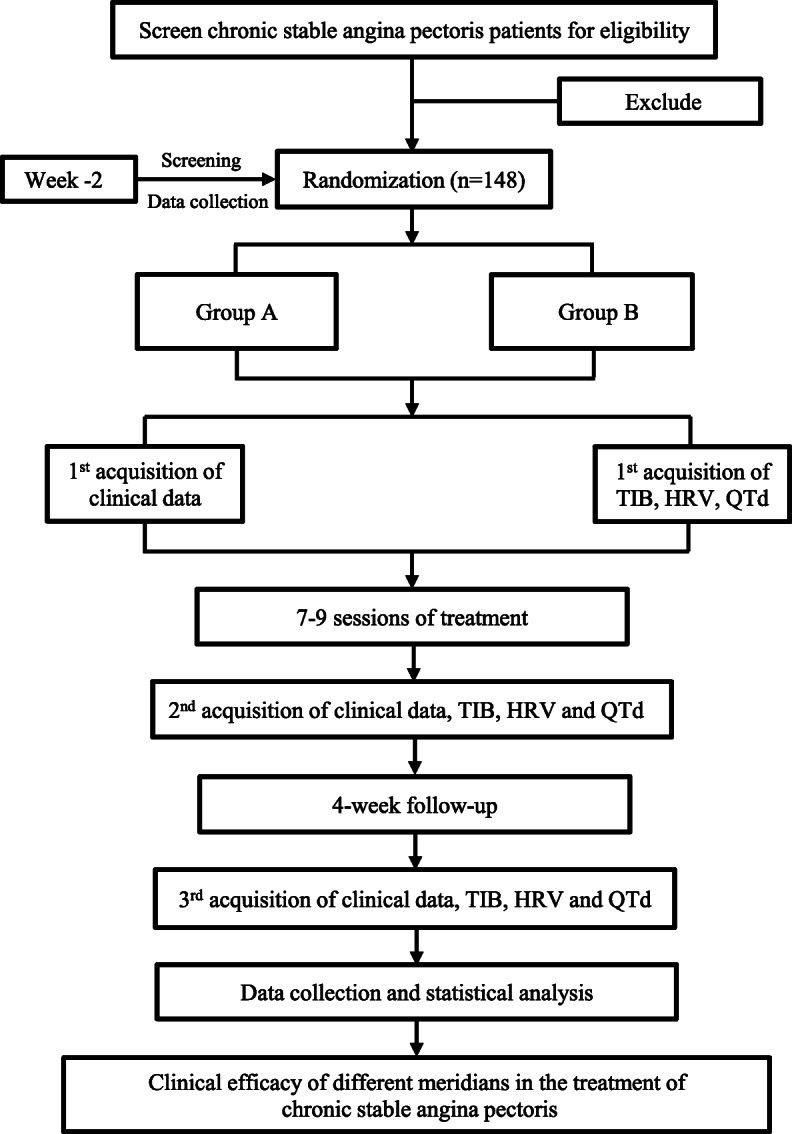
Flowchart of the study design

This is a two-arm, double-center, randomized controlled trial. A total of 148 CSAP patients will be enrolled and randomly assigned to two groups through central randomization in a 1:1 ratio. This trial will include a 2-week screening period, a 4-week treatment period, and a 4-week follow-up period. Outcome assessments will be performed at the baseline, the end of treatment, and the end of follow-up (see Fig. 1 for the details of the study schedule).

**Fig. 2:**
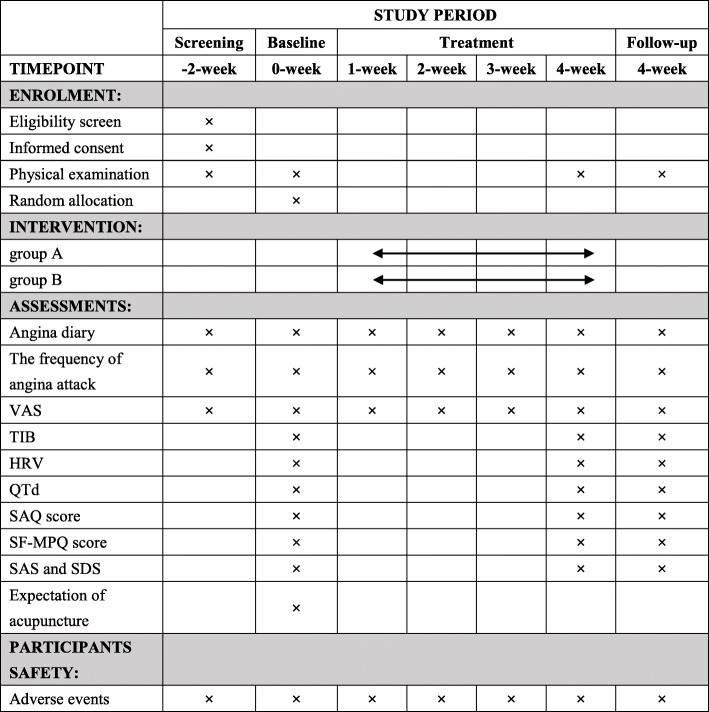
Standard Protocol Items: Recommendations for Interventional Trials (SPIRIT) schedule. VAS, visual analog scale; TIB, total ischemia burden; HRV, heart rate variability; QTd, QT dispersion; SAQ, score of Seattle Angina Questionnaire; SF-MPQ, Short-Form of McGill Pain Questionnaire; SAS, self-rating anxiety scale; SDS, self-rating depression scale

The reporting of this trial is conducted according to the Standard Protocol Items: Recommendations for Intervention Trials (SPIRIT) guidelines [19] (Fig. 2). The Consolidated Standards of Reporting Trials (CONSORT) and the Standards for Reporting Interventions in Clinical Trials of Acupuncture (STRICTA) have been used as frameworks of methodology for the design of this protocol [20].

### Participants

The CSAP patients will be recruited from two clinical centers, including the Hospital of Chengdu University of Traditional Chinese Medicine (CDUTCM) and the First Affiliated Hospital of Zhejiang Chinese Medical University (ZCMU).

All CSAP patients will undergo a careful physical examination including electrocardiogram (ECG), echocardiographic tissue Doppler imaging (TDI), blood pressure test, blood sugar test, routine test (blood, urine, and stool), biochemical blood test (ALT, AST, BUN, Scr), myocardial zymogram test (LDH, α-HBDH, CK, CKMB), serum immunoglobulin E (IgE), myoglobin (Myo), and cardiac troponin l (cTnl) before inclusion.

### Recruitment strategy

Participants will be recruited from the outpatient and inpatient departments of Cardiology in the Hospital of CDUTCM and the First Affiliated Hospital of ZCMU from October 2019 to August 2021.

The recruitment strategies include advertisements in the post, leaflets, the website of CDUTCM (http://cdutcm.edu.cn/), and ZCMU (https://www.zcmu.edu.cn/).

### Diagnostic criteria

The diagnostic criterion bases on the Guideline for Diagnosis and Treatment of stable coronary artery disease (SCAD) issued by Chinese Society of Cardiology, Chinese Medical Doctor Association of Cardiovascular Physicians and Editorial Committee of Chinese Journal of Cardiology in 2018 [21].

### Inclusion criteria

The patients will be included if they meet the following 6 items: (1) match the diagnostic criteria of SCAD; (2) meet the criteria of Class I or Class II according to the Canadian Cardiovascular Society (CCS) angina pectoris severity grade [22]; (3) age from 45 to 70 years; (4) have experienced angina attack in the last 3 months, and the attack frequency ≥ twice a week in the most recent month; (5) have a standardized medication history at least 2 months before enrolment (sustaining the same medication, usage, and dose), the basic therapeutic medication retains to one or more of the following: β-blockers ACEI, ARB, and antiplatelet medications and statins; and (6) have signed the informed consent.

### Exclusion criteria

The patients will be excluded if they match the following 9 items: (1) be accompanied with acute myocardial infarction, severe arrhythmia (advanced atrioventricular block, ventricular tachycardia, supraventricular tachycardia, frequent premature beats); (2) be accompanied with severe psychiatric illness; (3) fail to control hypertension (systolic blood pressure ≥ 140 mmHg and/or diastolic blood pressure ≥ 90 mmHg) or diabetes mellitus (HbA1 above 7% in last 3 months); (4) have severe digestive, urinary, respiratory, hematological, nervous, or endocrine system diseases; (5) be pregnant or during lactation or intending to be pregnant in the 6 months; (6) have received acupuncture therapy due to cardiovascular illness in the last 1 month; (7) have the bleeding tendency and dermatitis; (8) be in the allergic constitution and be allergic to adhesive tape; or (9) be participating in any other clinical trials.

### Sample size

In this study, we assume that H0 represents the clinical effect of Group A is the same as Group B; H1 represents the effect of Group A is not the same as Group B.

According to the formula: $n=\frac{2{\sigma}^2}{{\left(\mu 1-\mu 2\right)}^2}\times {\left({\mu}_{\alpha /2}+{\mu}_{\beta}\right)}^2$ [23]. In this study, assuming α = 0.05 (both sides), 1-β = 0.80. Based on our previous study [12], *μ*1 is the improvement of the frequency of angina attacks in the acupoints on the disease-affected meridian group, and *μ*2 is the improvement of the frequency of angina attacks in the acupoints on the non-affected meridian group. With *μ*1 = 5.57 and *μ*2 = 3.14, σ is estimated to be 5, and the sample size is calculated as 67 per group. Considering a dropout rate of 10%, we concluded that the sample size is 73.6 per group, and each group needs 74 cases after taking an integer. Therefore, a total of 148 CSAP patients will be finally recruited.

### Randomization

To guarantee randomization concealment adequately, and not be influenced either by the acupuncturists or the participants, randomization will be performed by a clinical information management system (Beijing Bioknow Information Science & Technology Co. Ltd., China). The investigators are trained to apply for randomization through text messages, online websites, or telephone application. When randomization information (including the participants’ name, gender, and date of birth) is sent to the randomization center, the random numbers and group assignment will immediately be delivered to the acupuncturists.

### Blinding

It is difficult to blind acupuncturists in acupuncture clinical trials. However, it is feasible to conceal the patients and group assignments from outcome assessors and statistical analysts. Patients are told that they will receive one of 2 effective acupuncture interventions after enrollment, and during the acupuncture treatment, patients in different groups will be in separate compartments to avoid communication. Outcome assessors and statisticians will be blind to the group allocations.

### Intervention

All patients in the two groups will receive a total of 7–9 acupuncture sessions (at least 7 sessions) during 4 weeks. By intradermal needle, acupuncture stimulation will last for 48–72 h in each session with a 1-day interval between each session.

**Fig. 3:**
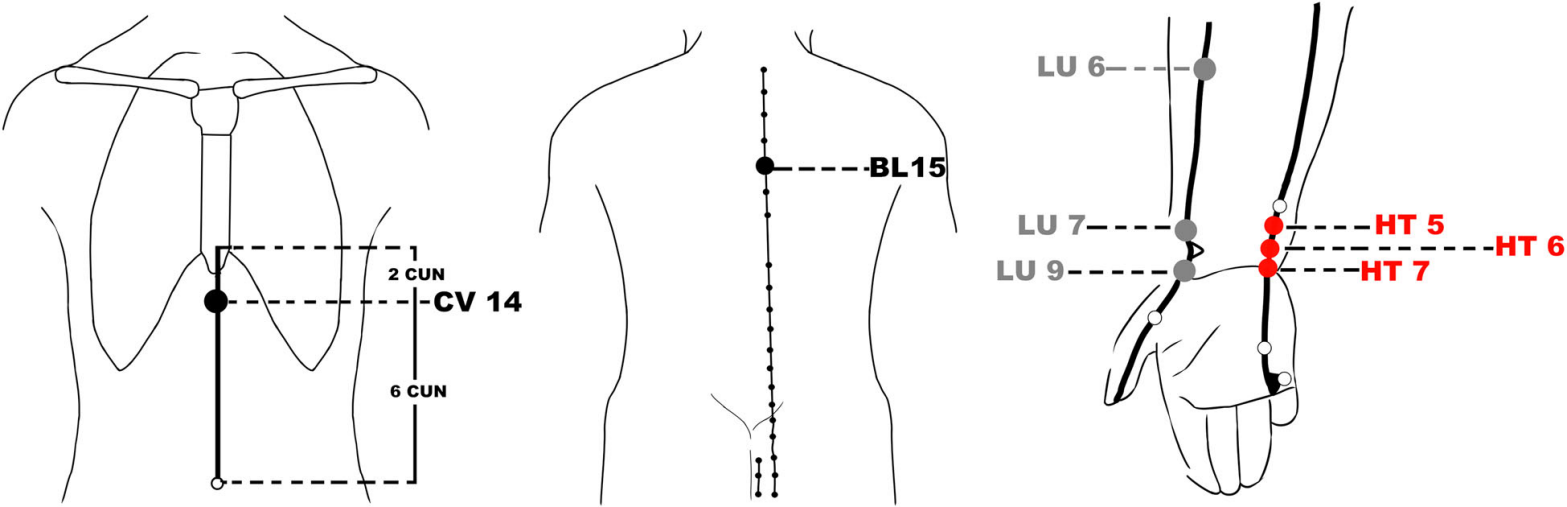
Locations of Acupoints. CV14 (*Juque*), on the anterior meridian line of the upper abdomen,6 cun above the navel. BL15 (*Xinshu*), on the back,1.5 cun on the lateral to the lower border of the spinous process of the 5th thoracic vertebra. HT7 (*Shenmen*), on the wrist, at the ulnar end of the transverse crease of the wrist, in the depression on the radial side of the tendon of the flexor carpi ulnaris. HT6 (*Yinxi*), on the palmar side of the forearm, the point is on the radial side of the tendon of the flexor carpi ulnaris,0.5 cun above the transverse crease of the wrist. HT5 (*Tongli*), on the palmar side of the forearm, the point is on the radial side of the tendon of the flexor carpi ulnaris,1 cun above the transverse crease of the wrist. LU9 (*Taiyuan*), on the radial side of the transverse crease of the wrist, where the radial artery pulsates. LU7 (*Lieque*), on the radial margin of the forearm,1.5 cun above the transverse crease of the wrist, between the brachioradial muscle tendon and the long abductor muscle tendon of thumb. LU6 (*Kongzui*), on the radial palmar side of the forearm, on the line connecting *Chize* (LU5) with *Taiyuan* (LU9),7 cun above the transverse crease of the wrist.

The acupoint prescription will be composed of the basic acupoints and additional acupoints. The basic acupoints include bilateral *Xinshu* (BL15) and *Juque* (CV14), which are close to the heart. The basic acupoints will be used for the patients in both groups. The additional acupoints are all on the forearm, which are far from the heart relatively. For group A, 3 additional acupoints on the Heart Meridian will be selected, including bilateral *Shenmen* (HT7), *Yinxi* (HT6), and *Tongli* (HT5). For group B, 3 additional acupoints on the Lung Meridian will be selected, including bilateral *Taiyuan* (LU9), *Lieque* (LU7), and *Kongzui* (LU6) (Fig. 3).

The intradermal needle (0.2 mm in diameter and 1.5 mm in length or 0.2 mm in diameter and 1.2 mm in length, Seirin Pyonex®; Seirin Corporation, Shizuoka, Japan) will be inserted and retained in each acupoint for 48–72 h (at least retained for 48 h). During the retaining period of the intradermal needle, patients will be requested to press the needle perpendicularly for 3 to 4 times every 24 h, and each time lasts for around 30 s. The manipulation will be performed according to the National Standard of the People’s Republic of China “Standardized Manipulations of Acupuncture and Moxibustion-part 8: Intradermal needle” (GB/T 21709.8-2008) [24].

### Basic treatment

The basic treatment for CSAP includes health education and primary medications, which will be performed from the baseline to the end of the follow-up period. Health education with CSAP aims to help the patients understand and master the basic knowledge of CSAP, such as risk factors, diagnosis, prevention, daily medication, and emergency treatment methods. Basic medications include β-blockers, ACEI, ARB, and antiplatelet medications and statins. Patients can choose one or several of the four medications as maintenance therapy according to cardiologists’ advice.

### Outcomes

#### Primary outcome

In this trial, the primary outcome is the frequency of angina attacks from baseline to 4 weeks after inclusion. The frequency of angina attacks will be recorded in the angina diary. The angina diary will be requested to complete and return to assessors in time by paper diary, emails, short messages, or telephone calls throughout the whole 10 weeks observation period.

#### Secondary outcomes

The secondary outcomes include: (1) the frequency of angina attacks from baseline to 4 weeks after the acupuncture treatment, (2) the pain intensity of angina which is assessed by visual analog scale (VAS), (3) total ischemia burden (TIB) and heart rate variability (HRV) which are recorded by 24-h dynamic electrocardiogram (DCG), (4) QT dispersion (QTd) which is recorded by ECG, (5) the score of Seattle Angina Questionnaire (SAQ), (6) the score of Short-Form of McGill Pain Questionnaire (SF-MPQ), (7) the score of self-rating anxiety scale (SAS), (8) the score of self-rating depression scale (SDS), and (9) the expectation values of acupuncture treatment which is composed of 7 questions and designed by our team. The VAS score will be extracted from the angina diaries. The second to eighth secondary outcomes will be evaluated at baseline, at the end of acupuncture treatment and at the end of follow-up. Moreover, the expectation of acupuncture treatment in each patient will be evaluated at baseline.

### Patient safety

To guarantee the safety of patients, acupuncture treatment will be performed in the cardiology department where cardiovascular specialists and first-line physicians can participate in emergency timely. All adverse events (AEs) such as pain, allergy, bleeding, hematoma, and fainting should be recorded in the case report form (CRF) carefully. Serious adverse events (SAE) such as death or life-threatening events should immediately be reported to the principal investigator.

### Practitioners training and quality control

All researchers involved in this trial must attend training classes to ensure all practices at each center are generalized and standardized according to the standard operating procedures (SOP). All the acupuncture manipulation will be performed by two licensed acupuncturists with clinical experience over 3 years. The acupuncturists should pass the examination on the SOP of acupuncture intervention before participating in this trial. In addition to the monthly inspection carried out by the research team, a specialized project supervision team will be established by the Clinical Research Center for Acupuncture and Moxibustion of Sichuan Province, and responsible for monitoring the study every 3 months.

### Data management

Data will be managed jointly by the two research centers. All the data will be recorded with printed and electronic case report forms CRFs. Only outcome assessors have access to CRFs and will perform the double-data entry.

### Data analysis

All analyses will be strictly conducted according to the intention-to-treat (ITT) principle. Continuous variables will be described with means and standard deviation (SD) or median, maximum, minimum, P25, and P75. Categorical variables will be described with numbers and percentages (%).

For the frequency of angina attacks from baseline to 4 weeks after inclusion, if the distribution conforms to the approximately normal distribution, analysis of covariance (ANCOVA) will be used. Since this study is a multicenter clinical trial, the center effects will be included in the analysis model. Age, gender, duration of the disease, and study site will be included in the model as covariates. Considering clustering effects of the number of angina attacks per patient, a hierarchical Poisson regression will be conducted exploratively. Separate ANCOVA analyses will be performed for secondary outcomes. The incidence of AEs in both groups will be compared with a Poisson regression model in which the number of participants with AEs is an independent variable and the group is a dependent variable.

Missing data will be imputed by multiple imputation. A sensitivity analysis using a pattern-mixture model will be conducted to assess the robustness of the results.

For all statistical analyses, Statistical Analysis System version 9.4 software (SAS Institute Inc., Cary, North Carolina) will be used. A *P* value of less than 0.05 (*α* value of 0.05, two-sides) will be considered statistically significant.

## Discussion

This is the first study to evaluate the efficacy of acupuncture with intradermal needle for treating CSAP at different acupoint prescriptions. The results might deepen our knowledge on the theory of Meridian-Viscera Association and provide reference for the selections of acupoint prescriptions and acupuncture modality so as to develop better practice for acupuncture treating CSAP.

### The theory of Meridian-Viscera Association

As an important component of the acupuncture theoretic system, the Meridian-Viscera Association theory emphasizes the close relationship between meridians and viscera in physiological function, pathological changes, diagnosis, and treatment. In detail, viscera physiological functions and pathological changes will manifest in the corresponding meridians or the acupoints [25], and the viscera disorders can be treated with the involved meridian and acupoints [26]. That means gastric disorders can manifest on the Stomach Meridian and be treated with the acupoints on Stomach Meridian. Cardiovascular disease can reflect on the Heart/Pericardium Meridian and be treated with the acupoints on the Heart /Pericardium Meridian. A number of modern studies have confirmed the relatively specific relationship between meridians and viscera and the modulating effect of meridians/acupoints for the corresponding viscera functions [27, 28]. For example, our previous study found that puncturing at the acupoints on the Stomach Meridian can effectively improve the symptoms and QoL of the functional dyspepsia (FD) patients compared to puncturing at sham acupoints or the acupoints on the Gallbladder Meridian [29].

The CSAP pertains to heart-related diseases. Acupuncture treatment can be performed on the meridians/acupoints which is related to the heart. In light of the traditional Chinese acupuncture theory, the Heart Meridian is directly related to the heart, and the Lung Meridian is indirectly connected to the heart. The acupoints on these two meridians can all be used for cardiovascular diseases. Therefore, this study tries to compare the therapeutic effects of the acupoints on the Heart Meridian (direct-related meridian) and the acupoints on the Lung Meridian (indirect-related meridian) for treating CSAP.

### Acupoints selection

In clinical practice, selecting the local points and distal points simultaneously is the basic principle for developing acupoint prescriptions. In this study, the local acupoints, including *Xinshu* (BL15, the Back-shu point of the heart) on the back and *Juque* (CV14, the Front-mu point of the heart) on the chest, will be selected as the basic points because they are close to the heart and are the most commonly used local acupoints for cardiovascular diseases. For the distal acupoints, 3 acupoints on the Heart Meridian or on the Lung Meridian will be selected respectively as the additional acupoints. They all locate on the forearm, which is far from the heart. They are the *Shenmen* (HT7), *Yinxi* (HT6), and *Tongli* (HT5) on the Heart Meridian for group A and the *Taiyuan* (LU9), *Lieque* (LU7), and *Kongzui* (LU6) on the Lung Meridian will be selected respectively for group B. According to the literature analysis [16, 28], these 6 acupoints were all used for treating cardiovascular diseases. Among them, *Shenmen* (HT7) and *Taiyuan* (LU9) are the Yuan-source points of the Heart Meridian or the Lung Meridian, respectively; *Tongli* (HT5) and *Lieque* (LU7) are Luo-connecting points of the Heart Meridian or the Lung Meridian, respectively; and *Yinxi* (HT6) and *Kongzui* (LU6) are Xi-cleft points of the Heart Meridian or the Lung Meridian, respectively.

### Acupuncture modality selection

Intradermal needle is a kind of special acupuncture needle for superficial puncture, which could be inserted perpendicularly into the skin with a tiny needle and be fixed with a piece of adhesive tape for 1 to 3 days to produce a gentle continuous stimulation on acupoint [30]. Compared with the filiform needle, the intradermal needle has more convenient operation, less pain, and fewer adverse reactions [17, 18]. So the intradermal needle has recently been widely used for chronic diseases such as chronic tension-type headaches and insomnia [31, 32]. In addition, a small sample size clinical study also indicated that the intradermal needle could significantly improve the clinical symptoms of CSAP [33]. Therefore, considering the simplicity of the operation and the continuity of stimulation, the intradermal needle has been selected as the acupuncture modality in this study.

### Basic treatments with medicines

The guidelines from the Chinese Society of Cardiology and the American College of Cardiology/American Heart Association (ACC/AHA) hold that the basic treatment of CSAP mainly includes health education and primary drugs [21, 34]. So in this study, health education and medications are selected as the basic treatment for the patients with CSAP.

In this study, all patients are recommended to modify their lifestyles and control unhealthy habits that might affect diseases, such as limiting alcohol consumption, controlling weight, and quitting smoking. Health education is beneficial for the patients to be aware of the disease, to correct unhealthy behaviors and eating habits in daily life, and to avoid various accidents or adverse conditions during treatment and improve the safety of treatments.

Based on the guidelines [21, 34] and cardiologists’ recommendations, this study will select the four most commonly used medications as basic medications for treating CSAP, including β-blockers, ACEI, ARB, and antiplatelet medications and statins.

In conclusion, focuses on the theory of Meridian-Viscera Association, this study tries to investigate the efficacy differences in improving the symptoms of CSAP between two commonly used meridians (acupoints prescription), so as to provide evidence for the selections of acupoints and acupuncture modality. The results might be valuable for developing good practice for acupuncture treating CSAP.

### Trial status

This trial was registered on 9 September 2019 at the Chinese Clinical Trial Registry (Registration number: ChiCTR1900025804, the protocol version number: F2.0). The trial is currently in the stage of patient recruitment. The first patient was included on 11 October 2019. To date, 12 patients have been included. Affected by the outbreak of Corona Virus Disease 2019 (COVID-19), recruitment will be approximately completed before 30 August 2021 and the trial is estimated to finish in November 2021.

## Data Availability

This trial does not involve the storage of biological specimens. The data and materials during the current study are available from the corresponding author on reasonable request.

## Abbreviations

CSAP: Chronic stable angina pectoris
CAD: Coronary artery disease
Qol: Quality of life
ACEI: Angiotensin-converting enzyme inhibitors
ARB: Angiotensin II receptor blockers
RCT: Randomized controlled trial
dPRP: Difference in pressure-rate-product between rest and maximum exercise
SPIRIT: Standard Protocol Items Recommendations for Intervention Trials
CONSORT: The Consolidated Standards of Reporting Trials
CDUTCM: Chengdu University of Traditional Chinese Medicine
ZCMU: Zhejiang Chinese Medical University
ECG: Electrocardiogram
TDI: Echocardiographic tissue Doppler imaging
IgE: Serum immunoglobulin E
Myo: Myoglobin
cTnl: Cardiac troponin l
SCAD: Stable coronary artery disease
CCS: Canadian Cardiovascular Society
VAS: Visual analog scale
TIB: Total ischemia burden
HRV: Heart rate variability
DCG: Dynamic electrocardiogram
QTd: QT dispersion
SAQ: Score of Seattle Angina Questionnaire
SF-MPQ: Short-Form of McGill Pain Questionnaire
SAS: Self-rating anxiety scale
SDS: Self-rating depression scale
AEs: Adverse events
SAE: Serious adverse events
CRF: Case report form
SOP: Standard operating procedures
ITT: Intention-to-treat
SD: Standard deviation
ANCOVA: Analysis of covariance
SAS: Statistical Analysis System
FD: Functional dyspepsia
ACC/AHA: The American College of Cardiology/American Heart Association
COVID-19: Coronavirus disease
